# Successive epidemic waves of cholera in South Sudan between 2014 and 2017: a descriptive epidemiological study

**DOI:** 10.1016/S2542-5196(20)30255-2

**Published:** 2020-12-02

**Authors:** Forrest K Jones, Joseph F Wamala, John Rumunu, Pinyi Nyimol Mawien, Mathew Tut Kol, Shirlee Wohl, Lul Deng, Lorenzo Pezzoli, Linda Haj Omar, Justin Lessler, Marie-Laure Quilici, Francisco J Luquero, Andrew S Azman

**Affiliations:** a Department of Epidemiology, Johns Hopkins Bloomberg School of Public Health, Baltimore, MD, USA; b World Health Organization, Juba, South Sudan; c Republic of South Sudan Ministry of Health, Juba, South Sudan; d World Health Organization, Geneva, Switzerland; e World Health Organization, Brazzaville, Republic of Congo; f Enteric Bacterial Pathogens Unit, Institut Pasteur, Paris, France; g Epicentre, Geneva, Switzerland; h Médecins Sans Frontières, Geneva, Switzerland

## Abstract

**Background:**

Between 2014 and 2017, successive cholera epidemics occurred in South Sudan within the context of civil war, population displacement, flooding, and drought. We aim to describe the spatiotemporal and molecular features of the three distinct epidemic waves and explore the role of vaccination campaigns, precipitation, and population movement in shaping cholera spread in this complex setting.

**Methods:**

In this descriptive epidemiological study, we analysed cholera linelist data to describe the spatiotemporal progression of the epidemics. We placed whole-genome sequence data from pandemic *Vibrio cholerae* collected throughout these epidemics into the global phylogenetic context. Using whole-genome sequence data in combination with other molecular attributes, we characterise the relatedness of strains circulating in each wave and the region. We investigated the association of rainfall and the instantaneous basic reproduction number using distributed lag non-linear models, compared county-level attack rates between those with early and late reactive vaccination campaigns, and explored the consistency of the spatial patterns of displacement and suspected cholera case reports.

**Findings:**

The 2014 (6389 cases) and 2015 (1818 cases) cholera epidemics in South Sudan remained spatially limited whereas the 2016–17 epidemic (20 438 cases) spread among settlements along the Nile river. Initial cases of each epidemic were reported in or around Juba soon after the start of the rainy season, but we found no evidence that rainfall modulated transmission during each epidemic. All isolates analysed had similar genotypic and phenotypic characteristics, closely related to sequences from Uganda and Democratic Republic of the Congo. Large-scale population movements between counties of South Sudan with cholera outbreaks were consistent with the spatial distribution of cases. 21 of 26 vaccination campaigns occurred during or after the county-level epidemic peak. Counties vaccinated on or after the peak incidence week had 2·2 times (95% CI 2·1–2·3) higher attack rates than those where vaccination occurred before the peak.

**Interpretation:**

Pandemic *V cholerae* of the same clonal origin was isolated throughout the study period despite interepidemic periods of no reported cases. Although the complex emergency in South Sudan probably shaped some of the observed spatial and temporal patterns of cases, the full scope of transmission determinants remains unclear. Timely and well targeted use of vaccines can reduce the burden of cholera; however, rapid vaccine deployment in complex emergencies remains challenging.

**Funding:**

The Bill & Melinda Gates Foundation.

## Introduction

The seventh cholera pandemic, first recognised in 1961, continues to plague populations without sufficient access to safe water and sanitation across the world. Although estimates of the global burden are uncertain, more than 140 000 suspected cholera cases are reported annually from sub-Saharan Africa.^1^ Many of these cases occurred in areas with poor infrastructure including peri-urban slums, rural areas dependent on surface water, and areas with complex emergencies. Universal access to water and sanitation would probably eliminate cholera transmission but is unlikely given the pace of progress and financial commitments.^2^

In 2017, the WHO-led Global Task Force on Cholera Control developed a roadmap to end cholera by 2030, and in 2018, a resolution to end cholera was adopted at the 71st World Health Assembly.^3^ The roadmap calls for a geographically targeted approach to use scarce resources in areas with a high risk of cholera, requiring an in-depth understanding of transmission across endemic and epidemic settings. South Sudan is one of the 47 focal countries of this roadmap.

Pandemic *Vibrio cholerae* has been introduced into the African continent at least 12 times over the past 50 years.^4^, ^5^ Cholera cases have been reported throughout the continent, where in some highly endemic countries such as the Democratic Republic of the Congo and Nigeria, cases are reported annually often with strong seasonal patterns.^6^, ^7^ In most other sub-Saharan African countries, such as South Sudan, cholera occurs with less regularity. Before 2005, there were few reports of cholera in South Sudan; between 2005 and 2009, annual epidemics were reported; then no cholera cases were reported until 2014.^8^ The South Sudanese civil war began in December, 2013, leading to damaged essential infrastructure and displacement of hundreds of thousands of individuals.

**Research in context**

**Evidence before this study**

We searched PubMed for articles published in English between Jan 1, 1970, and Sept 18, 2020, using the search terms “cholera”[title] AND (“epidemic” OR “outbreak”) AND (“Africa” OR “emergency” OR “conflict”) AND (“molecular” OR “sequencing” OR “rain” OR “precipitation” OR “mobility” OR “movement” OR “vaccine” OR “WASH”). We also downloaded reported cases and deaths from the Global Health Observatory Data repository on all outbreaks in the geographical area of South Sudan.Previous studies have shown associations between epidemic cholera and complex emergencies (eg, Yemen, Haiti, and Democratic Republic of the Congo), mass population movement (eg, Senegal), and precipitation (eg, Yemen and Haiti); however, it is unclear how these factors interact to shape cholera epidemics. Oral cholera vaccination campaigns have only recently been used both in response to epidemics and preventatively, but their effect at the population level has not been well described.

**Added value of this study**

Our study helps explain the complex story of three epidemic waves of cholera in South Sudan that began soon after the beginning of the civil war. We showed how each wave began in the capital city of Juba at the start of the rainy season and spread to different areas of the country. Using molecular data, isolates from all 4 years were found to be from the same clonal origin. We presented evidence, including the analyses of previously published whole-genome sequences, that cholera was probably introduced in 2013 or 2014 from Uganda or the Democratic Republic of the Congo and might have been reintroduced through multiple cross-border transmission events. We identified that large-scale population displacement and movement partially explained the differences in the number of cases between years and transmission during the dry season of the 2016–17 wave. Furthermore, we describe 36 vaccination campaigns providing over 2 million doses of cholera vaccine: we found that early vaccination might have reduced local outbreak sizes, although most vaccination activities occurred after the peak week of cases.

**Implications of all the available evidence**

Our research highlights the need for regional-level responses to curb outbreaks of cholera in humanitarian settings. In addition to oral cholera vaccine campaigns and interventions to improve water, sanitation, and hygiene in these vulnerable settings, controlling cholera in nearby countries that have the potential to introduce cholera might be an effective additional strategy to reduce cases. Increased sampling of *Vibrio cholerae* from humans and from environmental samples combined with whole-genome sequencing can help us better understand how cholera spreads across borders and maintains itself in the environment. Expanding the use of sequencing and bioinformatics in more countries with *V cholerae* transmission could lead to improved global representativeness of sequences and integration of results into local decision-making processes. Although increased precipitation has been linked to increased cholera transmission in many settings, we found that it was associated with the onset of outbreaks, but not with increases in transmission once an outbreak has begun. Although our study adds to the evidence on the role of population movement in cholera outbreaks, improved methods for measuring population movement within and between countries during complex emergencies is needed to better characterise these relationships.

Three successive epidemic waves of cholera occurred in South Sudan between 2014 and 2017, leading to tens of thousands of cases and hundreds of deaths, many among internally displaced individuals.^9^, ^10^, ^11^ Each wave varied greatly by geographic extent, timing, and magnitude and were separated by periods with few to no reported cases. It is unclear whether each outbreak was the result of a new introduction of *Vibrio cholerae* O1 or whether cholera was present but undetected between outbreaks.

Here, using detailed cholera case data collected from 2014 to 2017, we describe the key features of the successive epidemic waves and explore how precipitation, population displacement, and vaccination campaigns might explain the differences in cholera incidence and spatial spread between waves.

## Methods

### Cholera surveillance system

The Ministry of Health of the Republic of South Sudan, in collaboration with local health authorities, implemented cholera surveillance through both the Integrated Disease Surveillance and Response and Early Warning Alert and Response Network with support from WHO and Health Cluster. Linelist data were collected from all sites treating patients with cholera across South Sudan, including cholera treatment centres and oral rehydration points, from June 2014, to December 2017. Before the confirmation of cholera, a suspected case was defined as any patient aged at least 5 years who developed severe dehydration or died from acute watery diarrhoea. After cholera was confirmed within a county, a suspected cholera case was any patient aged at least 2 years who developed acute watery diarrhoea. A confirmed case was a suspected case with culture-confirmed *V cholerae* O1. We use the term epidemic waves to describe an increase and decrease of reported cholera cases at the national level, which is a different concept to the previously described waves of the seventh pandemic.^12^

### Laboratory analysis

When suspected cases were first reported in a county, stool specimens from some of the suspected cases were sent to the National Public Health Laboratory (Juba, South Sudan) for culture confirmation.^13^ Additional isolates were collected from suspected cases and tested by culture at the National Public Health Laboratory in 2015 in a vaccine effectiveness study.^14^ Overall, 150 isolates from South Sudan (and ten from Uganda) were sent to the French National Reference Center for Vibrios and Cholera (Institut Pasteur, Paris, France) for serogroup and serotype identification, antibiotic resistance pattern testing (disk diffusion following Comité de l’Antibiogramme de la Société Française de Microbiologie 2013 standards for Enterobacteriaceae), measurment of minimum inhibitory concentrations of nalidixic and ciprofloxacin by Etests (AB bioMérieux, Solna, Sweden), amplification and sequencing of the genes encoding DNA gyrase (*gyrA* and *gyrB*) and topoisomerase IV (*parC* and *parE*), genotyping (*rstR*, *ctxB*, *tcpA*, *gyrA*, *gyrB*, *parE*, and *parC*),^15^ and multi-locus variable number tandem repeat analysis (MLVA).^4^, ^13^, ^16^ Isolates from each year of isolation were subjected to whole-genome sequencing, genomic data were placed in the full context of the seventh pandemic in a prior study.^4^

### Data sources

Given that few sites directly measured rainfall in South Sudan during the study period, we used remote sensing-derived estimates. We obtained average daily precipitation estimates from 2010–18 at the country, state, and county level from the Climate Hazards Group InfraRed Precipitation with Station Data dataset.

We used county-level population estimates from WorldPop. To better understand population movements as a result of conflict, we acquired mobility tracking data from the International Office of Migration. During the second round of mobility tracking (March to April 2018), International Office of Migration officers interviewed key informants (eg, local authorities, community leaders, and humanitarian partners) in 46 of 78 counties of South Sudan to estimate the number of individuals (internally displaced people and returnees) present in assessment areas. These counties include 21 of 34 cholera-affected counties accounting for 66% of suspected cholera cases across the study period. Key informants also provided information on the year when individuals arrived and from where they came.^17^

WHO collated data from all oral cholera vaccination campaigns done between 2014 and 2018. We attempted to collate data on water, sanitation, and hygiene (WASH) interventions but these data were not systematically reported.

### Data analysis

We calculated the weekly number of cases, attack rate, case–fatality risk, the proportion of cases in people younger than 5 years, and proportion who were men. For most cases, we relied on the self-reported date of symptom onset for each case; when this was not available, we used the date of health facility visit. Administrative vaccination coverage was calculated by dividing the total number of first-round doses delivered by the estimated target population size. To explore the potential effect of vaccination campaigns, we compared the attack rates of outbreaks where vaccine was used before the epidemic peak with those where it was used during or after the peak with Poisson regression models using the population size as an offset. To be conservative, we excluded campaigns where no cholera cases were reported during the same wave.

We generated a maximum likelihood tree from the 1210 publicly available *V cholerae* O1 whole genomes.^4^, ^5^, ^18^ This tree closely resembles the phylogeny of 1203 genomes from Weill and colleagues,^4^ but includes seven genomes from Uganda published separately.^18^ GenBank accession numbers for all 1210 sequences are in appendix 1.^19^ For each reference-based assembly (reference accession AE003852/AE003853), we masked recombinant sites manually) and using Gubbins version 2.3.4^20^ as previously described.^5^ We generated the tree from the filtered_polymorphic_sites FASTA output by Gubbins, which contained only the 10 098 variant sites in our alignment. We used IQ-TREE, version 1.6.10,^21^ with a generalised time reversible substitution model and 1000 bootstrap iterations.^22^ We rooted the tree on A6 by first constructing a tree containing an outgroup sequence (M66, accession CP001233/CP001234) and choosing the most ancestral non-outgroup strain. We visualised phylogenetic trees using FigTree version 1.4.4. For MLVA analysis, we defined a clonal complex as a group of isolates where each differs (in loci-specific copy number) from at least one other isolate in the complex at no more than one locus.

We used a distributed lag non-linear model (appendix 2 p 7)^23^, ^24^, ^25^ to investigate how accumulated rainfall over the previous 7 days (AR7D) is associated with changes in cholera transmission in Juba county, as characterised by the instantaneous basic reproductive number, R_0_(t). We first jointly estimated the serial interval and reproductive number for the period of epidemic growth for each wave using methods described by White and Pagano.^26^ R_0_(t) was calculated using previously described methods^27^ assuming a shifted γ distributed serial interval with a mean of 4·1 days and a variance of 16·6 days based on the estimates of the 2015 wave, which was largely confined to Juba county (appendix 2 p 9). We assumed that infection leads to complete immunity over the study period and that 10% of infections were reported as medically attended cholera cases.^28^ To account for overdispersion, we fitted a quasipoisson model with penalised splines transforming AR7D and lags up to 10 days. The results from this model provide an estimate of the relative R_0_(t) over 10 days following a day with a given value of AR7D compared with an AR7D of 0 mm.

We aggregated population movement data to the county level. The origin of returnees immigrating to South Sudan is presented at the country level. Data were most often available at the annual scale (eg, population movement for 2015–16 was evenly split between the 2 years). We calculated the expected number of cholera cases exported by one county to another by multiplying the attack rate for each county by the number of people leaving that area during the same year.

We used state and county geographical boundaries from 2014 for all analyses. All code used for the analysis are available online.

### Role of the funding source

The funders of the study had no role in study design, data collection, data analysis, data interpretation, or writing of the report. All authors had full access to all data in the study and were responsible for the final decision to submit for publication.

## Results

**Figure 1:**
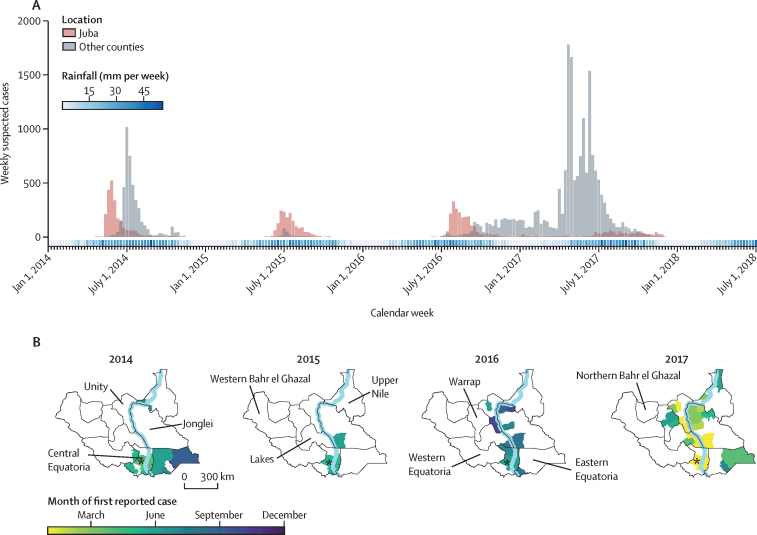
**Successive epidemic curves (A) and geographic spread of suspected cholera cases (B) in South Sudan between Jan 1, 2014, and July 1, 2018** For each year with cholera cases (2014, 2015, 2016, and 2017), county-level maps indicate the month when the first case was reported. Asterisks show Juba, the capital city of South Sudan. The light blue line shows the Nile river.

**Figure 2:**
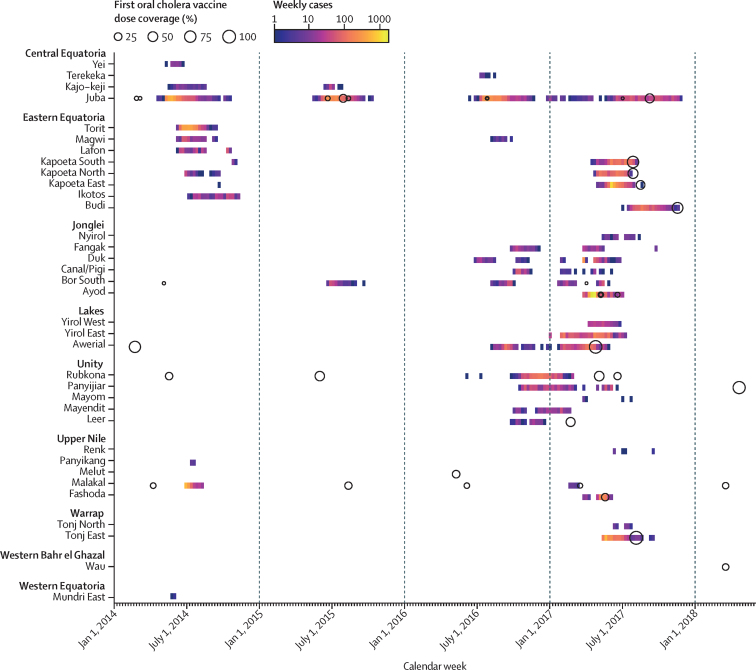
**Relative timing of oral cholera vaccine campaigns and county-level cholera outbreaks in South Sudan** Each circle represents a distinct oral cholera vaccination campaign and the proportion of the county-level population estimated to be vaccinated during the first round (ie, doses delivered divided by the estimated population size). Campaigns are marked on the week of the first round, and cases are marked at the week of reported symptom onset for each county.

After 3 years without cases, the Ministry of Health confirmed a cholera case in Juba county (Central Equatoria state) on April 28, 2014, soon after the start of the rainy season. Reports of cases increased in Juba county and in the neighbouring Eastern Equatoria state (figures 1, 2), where case–fatality risks reached as high as 5·9% in Lafon county (table). Cholera was then reported in the northeastern state of Upper Nile (1044 cases) where most cases came from internally displaced people outside of Malakal (South Sudan's second largest city). Over the course of 29 weeks, 13 counties reported 6389 suspected cholera cases (attack rate of 0·5 per 1000 population) and 139 cholera-related deaths (case–fatality risk of 2·2%; table). Among suspected cases, 1604 (25·1%) were children younger than 5 years and 3278 (51·3%) were men. Overall, 430 suspected cases from five different states underwent laboratory confirmation; 190 (44·2%) were confirmed positive for *V cholerae O1* by culture (appendix 2 p 2). The last confirmed and suspected case reported in 2014 was on Oct 27 (confirmed) and on Nov 13 (suspected) in Eastern Equatoria state.

**Table:** Cholera indicators in South Sudan overall and by county for the three epidemic waves

		**Population per 1000**	**Cases**			**Deaths**			**Attack rate (per 1000 population)**			**Case–fatality risk (%)**		
			Wave 1	Wave 2	Wave 3	Wave 1	Wave 2	Wave 3	Wave 1	Wave 2	Wave 3	Wave 1	Wave 2	Wave 3
South Sudan		11 654	6389	1818	20438	139	47	436	0·5	0·2	1·8	2·2	2·6	2·1
Central Equatoria														
	Juba	515	2229	1622	2928	40	45	37	4·3	3·2	5·7	1·8	2·8	1·3
	Kajo-keji	269	84	57	0	6	1	0	0·3	0·2	0·0	7·1	1·8	..
	Terekeka	190	0	0	15	0	0	5	0·0	0·0	0·1	..	..	33·3
	Yei	287	48	0	0	2	0	0	0·2	0·0	0·0	4·2	..	..
Eastern Equatoria														
	Budi	138	0	0	805	0	0	81	0·0	0·0	5·8	..	..	10·1
	Ikotos	103	297	0	0	5	0	0	2·9	0·0	0·0	1·7	..	..
	Kapoeta East	238	1	0	2105	0	0	26	0·0	0·0	8·9	0·0	..	1·2
	Kapoeta North	142	81	0	993	1	0	1	0·6	0·0	7·0	1·2	..	0·1
	Kapoeta South	107	14	0	1016	0	0	10	0·1	0·0	9·5	0·0	..	1·0
	Lafon	85	256	0	0	15	0	0	3·0	0·0	0·0	5·9	..	..
	Magwi	239	243	0	29	10	0	1	1·0	0·0	0·1	4·1	..	3·4
	Torit	149	2087	0	0	38	0	0	14·0	0·0	0·0	1·8	..	..
Jonglei														
	Ayod	199	0	0	3855	0	0	39	0·0	0·0	19·4	..	..	1·0
	Bor South	316	0	139	307	0	1	8	0·0	0·4	1·0	..	0·7	2·6
	Canal/Pigi	138	0	0	206	0	0	5	0·0	0·0	1·5	..	..	2·4
	Duk	93	0	0	542	0	0	36	0·0	0·0	5·8	..	..	6·6
	Fangak	155	0	0	261	0	0	2	0·0	0·0	1·7	..	..	0·8
	Nyirol	164	0	0	72	0	0	2	0·0	0·0	0·4	..	..	2·8
Lakes														
	Awerial	68	0	0	1197	0	0	13	0·0	0·0	17·6	..	..	1·1
	Yirol East	114	0	0	1496	0	0	67	0·0	0·0	13·1	..	..	4·5
	Yirol West	97	0	0	238	0	0	0	0·0	0·0	2·4	..	..	0·0
Unity														
	Leer	78	0	0	94	0	0	3	0·0	0·0	1·2	..	..	3·2
	Mayendit	89	0	0	226	0	0	5	0·0	0·0	2·6	..	..	2·2
	Mayom	179	0	0	9	0	0	4	0·0	0·0	0·1	..	..	44·4
	Panyijiar	72	0	0	612	0	0	30	0·0	0·0	8·5	..	..	4·9
	Rubkona	155	0	0	1176	0	0	10	0·0	0·0	7·6	..	..	0·9
Upper Nile														
	Fashoda	46	0	0	584	0	0	9	0·0	0·0	12·7	..	..	1·5
	Malakal	176	1035	0	19	22	0	0	5·9	0·0	0·1	2·1	..	0·0
	Panyikang	34	9	0	0	0	0	0	0·3	0·0	0·0	0·0	..	..
	Renk	199	0	0	10	0	0	0	0·0	0·0	0·1	..	..	0·0
Warrap														
	Tonj East	173	0	0	1611	0	0	42	0·0	0·0	9·3	..	..	2·6
	Tonj North	241	0	0	31	0	0	0	0·0	0·0	0·1	..	..	0·0
Western Bahr el Ghazal														
	Raga	81	0	0	1	0	0	0	0·0	0·0	0·0	..	..	0·0
Western Equatoria														
	Mundri East	83	3	0	0	0	0	0	0·0	0·0	0·0	0·0	..	..

Two suspected cases in the first wave did not have a county reported. No cholera cases were reported in Northern Bahr el Ghazal. Wave 1 was in 2014; wave 2 was in 2015; wave 3 was in 2016–17.

Following a period of 27 weeks with no suspected cases, the first confirmed case of the second wave was reported in Juba county on May 20, 2015. In 2015, over the course of 21 weeks, 1818 suspected cases (attack rate of 0·2 per 1000 population) and 47 deaths (case–fatality risk of 2·6%) were reported. Among suspected cases, 331 (18·2%) were younger than 5 years and 1004 (55·2%) were men. Nearly all suspected cases (1622 [89·2%]) came from Juba county between May 18 and Oct 15; the remaining 196 (10·8%) cases were reported in two nearby counties (figure 1B; table). Of the 132 tested samples from suspected cases in 2015, 43 (32·6%) were confirmed positive by culture. The last confirmed and suspected cases from the 2015 wave occurred in Juba county on Sept 11 (confirmed) and Oct 15 (suspected).

Following a period of 34 weeks with no suspected cases, South Sudan began to have its largest reported cholera outbreak in recent history, with 20 438 suspected cases (attack rate of 1·8 per 1000 population) and 436 deaths (case–fatality risk of 2·1%; table). Among suspected cases, 4450 (21·8%) were younger than 5 years and 9719 (47·6%) were men. On June 18, 2016, Juba county reported the first confirmed cholera case of the year within the country, later than the two previous years. In 2016, 139 (34·6%) of 402 suspected cases that were tested were confirmed. Confirmed cases occurred in Central Equatoria state, Eastern Equatoria state, Jonglei state, Lakes state, and Unity state (appendix 2 p 2). As rainfall decreased from October to December, 2016, no additional counties reported cholera.

Through the dry season (roughly December to March), counties along the Nile River continued to report suspected cases (figure 2). Beginning in January, 2017, outbreaks were observed in new areas of the Lakes, Unity, and Jonglei states. Ayod county (Jonglei state) had a particularly explosive outbreak with 3855 suspected cases and 39 deaths occurring between March 27 and July 9, 2017 (>250 cases per week) and 3276 cases over a 3-week period; many of these cases occurred in cattle camps, where nomadic pastoralists have little to no access to safe water and sanitation.^29^ As the rainy season began in 2017, suspected cases were detected in northeastern (Upper Nile state), southeastern (Eastern Equatoria state), and central (Warrap state) South Sudan. In 2017, 231 (51·0%) of 453 suspected cases that were tested across seven states were confirmed by culture. The last confirmed and suspected cases were reported on Aug 30 (confirmed) and Nov 29 (suspected), with no additional cases reported up to Nov 16, 2020.

In addition to case management, surveillance, and WASH interventions, 2 677 867 doses of oral cholera vaccine were administered in South Sudan between 2014 and 2018. There were 36 vaccine campaigns (six in 2014, six in 2015, four in 2016, 17 in 2017, and three in 2018) across 14 of the 34 cholera-affected counties (ten campaigns were in Juba county). The population reached during each campaign's first round (typically there were two rounds) ranged between 1926 and 206 521 people. Although the administrative coverage (number of doses delivered as a proportion of estimated population) of most campaigns in the targeted population was moderate to high (range 52–119%), the coverage at the county level was much lower. The median first-dose administrative coverage at the county level was 18% (IQR 4–41; figure 2).

Among 22 campaigns conducted reactively (ie, in response to cases), only five were before or during the county-specific peak week of cases. No reactive campaigns occurred in 2014 (figure 2; appendix 2 p 3). In 2015, one reactive campaign was during the peak week and two were after. In 2016 and 2017, one reactive campaign was before the peak, three were during, and 15 were after. Five campaigns took place after the last suspected case in a county epidemic, all in 2017. All five counties with vaccination campaigns in 2014 or 2015 reported cases during the 2016–17 wave, although it is unclear if these were in the same sub-county populations. Counties with campaigns occurring during or after the county-level epidemic peak had, on average, 2·2-times (95% CI 2·1–2·3) higher attack rates than those where vaccine campaigns occurred before the peak, even after adjusting for vaccine coverage and epidemic wave.

**Figure 3:**
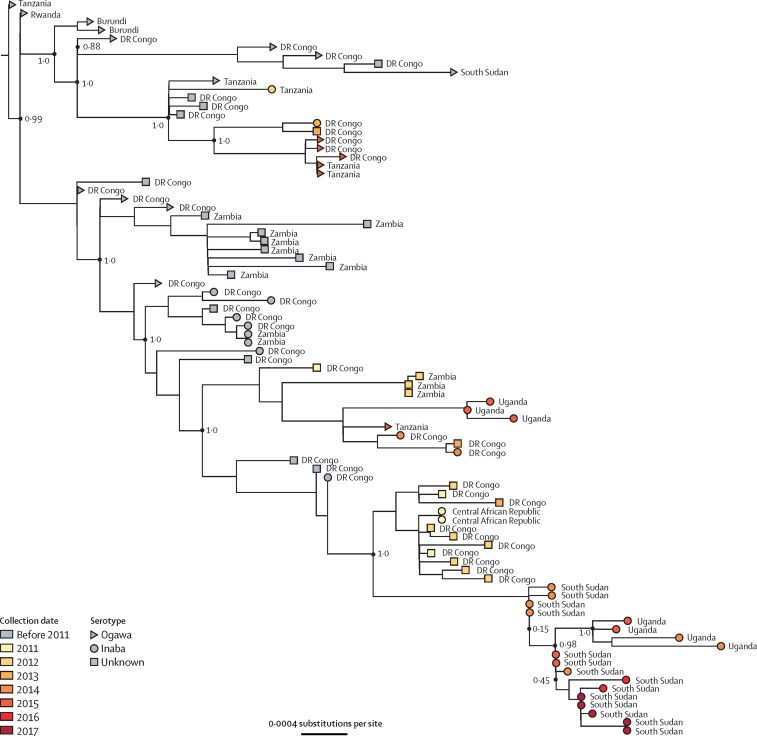
**Phylogenetic relatedness of** Vibrio cholerae **O1 El Tor isolates from South Sudan between 2014 and 2017** A subset of T10 lineage isolates published in Weill and colleagues,^5^ including 14 isolates from South Sudan, are combined with T10 lineage isolates from Uganda published in Bwire and colleagues.^18^ Bootstrap values at key nodes are labelled.

Results from phylogenetic analyses (figure 3; appendix 2 p 4), MLVA, and antibiotic resistance testing (appendix 2 p 5) all suggest that the bacteria circulating during the study period were nearly identical *V cholerae* O1 Inaba strains with the *ctxB1* genotype, sharing the characteristics of the 2014 isolates reported in a previous study.^13^ Our phylogenetic analysis looked more closely at previously published T10 lineage sequences from several African countries, including the 14 sequences from South Sudan within this clade.^4^, ^5^ We added seven recently published T10 sequences from Uganda in the same phylogenetic tree to better understand the relationship between *V cholerae* in these neighbouring countries.^18^ All 14 whole-genome sequences from South Sudan epidemics between 2014 and 2017,^5^ from patients in Central Equatoria state and Eastern Equatoria state, had very similar sequences. Across these 14 sequences, there are 21 polymorphic sites (about 3% of the diversity observed in the T10 lineage), with no more than ten SNPs between any two sequences (figure 3). These sequences all belonged to the T10 lineage, which has circulated in the Democratic Republic of the Congo and Uganda for more than 20 years (appendix 2 p 4) and is distinct from the T13 lineage recently reported in east and southern Africa and Yemen.^4^, ^30^ Four sequences from Uganda in 2014–15 cluster closely with all 14 sequences from South Sudan.

MLVA analysis on 150 *V cholerae* isolates collected from 12 different counties in seven different states in 2014 (46 isolates), 2015 (27 isolates), 2016 (42 isolates), and 2017 (35 isolates) showed that all isolates were from the same clonal complex (appendix 2 p 5). Ten isolates collected in Uganda in 2014 also belonged to the same clonal complex. Nearly all isolates had identical antibiotic resistance profiles, including resistance to colistin, nitrofurantoin, polymyxin B, streptomycin, sulfonamides, and co-trimoxazole (appendix 2 p 5).

More than 90% of average annual precipitation during 2010–18 occurred between April and November, both nationally and in Juba county (appendix 2 p 6). Rainfall in 2014–17 differed little from that in 2010–13, when no cholera was reported within the country, despite the 2014–16 El Niño event.^31^ With the exception of 2017, the rainy season preceded the detection of cases every year, with cases in Juba county typically reported in closest proximity (2–10 weeks) to the start of the heavy rains (appendix 2 p 6). In Juba county we found no significant association between accumulated rainfall over 7 days and the instantaneous basic reproductive number in the subsequent 10 days (appendix 2 p 10). Although we did not extend this analysis to all counties, it is noteworthy how this finding contrasts with those from analyses of cholera in Yemen using similar methods.^23^

Between 2014 and 2017, the International Office of Migration estimated that more than 760 000 individuals moved between counties, primarily due to the civil war in South Sudan or immigrated from a neighbouring country (appendix 2 p 11). In 2017, nearly two-times as many people immigrated than in 2014 (164 119 in 2017, 154 840 in 2015, 166 864 in 2016, and 274 965 in 2017). Most movement was between counties (553 689 people) with substantial international migration from Ethiopia (3025 people), Democratic Republic of the Congo (4653 people), Kenya (16 876 people), Sudan (87 175 people), and Uganda (95 370 people).

**Figure 4:**
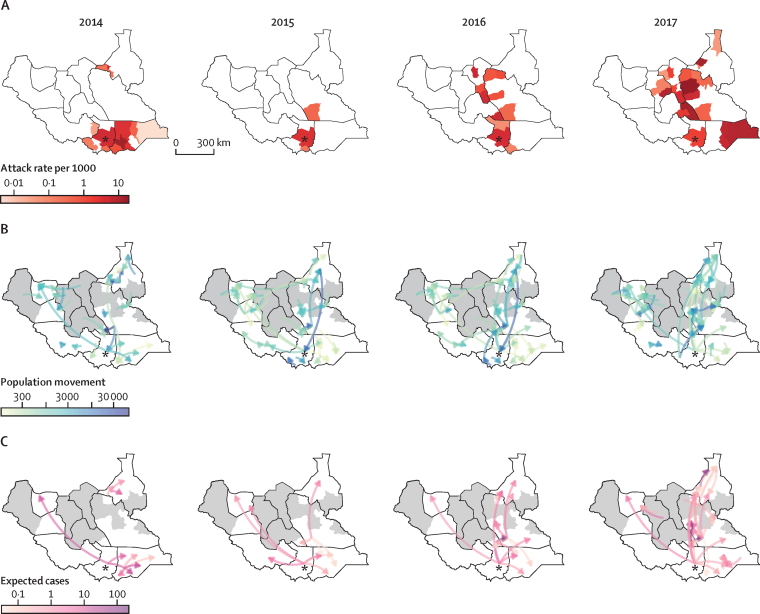
**County-level suspected case attack rate and population movement in South Sudan, 2014–17** (A) Attack rates per 1000 population. (B) Number and direction of internally displaced people and returnees moving between counties. (C) Expected number of exported cholera cases estimated by multiplying the attack rate of each county by the number of people leaving that county to another location for the same time period. log_10_ transformed colour scales are used for each map. Counties in grey were not assessed for arrival of internally displaced people or returnees. Arrows move to and from county-level centroids. Asterisks show Juba, the capital city of South Sudan. State borders are shown in black. Arrows with small values (<100 people or 0·01 expected cases) are not shown.

The movement of people out of counties reporting cholera was generally consistent with the year-to-year geographic extent of each wave (figure 4; appendix 2 p 12). In 2014, movement from Juba county, where the first cases were reported, was primarily eastward with little movement north. That same year, cholera was primarily reported in and around Central Equatoria state and in Eastern Equatoria state. In 2015, there was far less movement from Juba county outwards (66% fewer people leaving Juba county than in 2014), and the epidemic remained geographically limited. In 2016 and 2017, movement up and down the Nile River increased dramatically, including an increase in movement from Juba county northwards along the river. During this same period, cholera cases were reported from nearly every county along the Nile. In addition to internal migration, more than 110 000 returnees came from Kenya and Uganda, particularly in 2016–17 when both countries reported cholera cases (appendix 2 p 13).

## Discussion

In 2014–17, South Sudan had three distinct epidemic waves of cholera, all beginning in Juba county. Genotypic and phenotypic analyses indicate that despite periods of no reported cases between each wave, all were of the same clonal origin and most closely related to the *V cholerae* O1 circulating in Uganda and Democratic Republic of the Congo. Each wave occurred soon after the onset of the rainy season, although rainfall was not associated with increased transmission in Juba county. Movements of internally displaced people and returnees appeared consistent with spatial patterns of cholera, although we could not quantify this association because of the resolution of the data.

Cholera was probably introduced into South Sudan in 2013 or early 2014 through cross-border human population movement.^16^ It remains unclear whether *V cholerae* O1 remained in South Sudan between waves through undetected transmission (eg, insensitive surveillance or asymptomatic carriers), whether it persisted in the environment, or whether it was reintroduced. Given the magnitude of population movement between Uganda and South Sudan, reported cases in Uganda during the lull periods in South Sudan, increased surveillance efforts in South Sudan, and the genetic similarity between isolates from Uganda and South Sudan, repeated cross-border transmission events might be the most plausible explanation. This highlights the need for a regional approach to fighting cholera: efforts to slow an outbreak in one country could be undone by introductions from elsewhere.

For each wave, the first suspected cases in Juba county were detected shortly after the onset of the rainy season. Flooded infrastructure or changes in human behaviour around the time of the rainy season probably led to conditions favourable to the propagation of *V cholerae* O1 in Juba county. Notably, increases in rainfall once the rainy season had begun were not associated with increases in transmission in Juba, suggesting that rain might have played a role in triggering but not amplifying the outbreaks. In each wave, Juba county was the first county (except narrowly in 2016) to report suspected cases of cholera in the country and probably played an important role in maintaining cholera outbreaks in the country because of the size of the population and the substantial population movement in and out of the city.^29^ Juba county had more than 44% of weeks from 2014–18 with suspected cholera cases and a mean annual incidence of 2·6 per 1000, making it a clear burden hotspot, as defined by the Global Task Force on Cholera Control.^32^

Both increased human migration and transmission occurring during the dry season might partially explain why the 2016–17 outbreak spread more broadly across the country. In July, 2016, intense fighting began in Juba county, causing mass population displacement towards swampy areas along the Nile river and to Uganda. Simultaneously, the number of cases in Juba county was skyrocketing and cases began to be reported from a number of counties adjacent to the river.^33^ Cholera persisted during the dry season only in counties along the river. Though the reason for continued transmission remains unclear, most of these communities had not reported cholera cases in the preceding few years and presumably had low population-level immunity. The large geographic extent of cholera cases at the end of the dry season might have then created conditions where cholera transmission could be easily amplified as heavy rains arrived. Similar dynamics were noted in Yemen, where cholera expanded geographically during the dry season with cases exploding when rainfall increased.^23^

The widespread use of cholera vaccine (both reactively and pre-emptively) in South Sudan highlights some of the challenges in implementing campaigns, especially in humanitarian settings. Cholera control efforts during these outbreaks was continually hampered by conflicts and restricted access to areas with ongoing transmission. Of the 1 805 452 doses used reactively, only 4·1% were part of campaigns initiated before or during the peak of the epidemic in the target county. Because of the global shortage of vaccine supply, campaigns were often small in size and rarely covered the full population; only those deemed at the highest risk were targeted. Given the low coverage of cholera vaccine at the county level (though relatively high coverage in the target population), the spatial scale of the most reliable epidemiological data collected, and the low testing rate among suspected cases, the effect of vaccination on reducing incidence is unclear. Our simple analyses exploring the relative attack rates in places that had oral cholera vaccination campaigns before and after the peak provide hints of effects at the population level, but efforts to collect more spatially resolved incidence data combined with systematic lab testing of suspected cholera cases will allow for more precise quantification of vaccine effect.

This study comes with a number of limitations. We relied on reports of suspected cases from reporting health structures across the country. However, because of the instability in the region, some health centres might not have reported cases, and some people might not have sought care for even severe disease. Furthermore, as previous work has shown,^34^, ^35^ only a proportion of suspected cases are likely to be true cholera cases. Isolates that underwent whole-genome sequencing came exclusively from the southern portion of the country and were not geographically representative. However, MLVA analyses and the antibiotic sensitivity testing suggest similarity of strains across the country. Although interventions such as chlorination of public water sources, hygiene promotion, and enforcement of sanitation standards in public areas did occur, we were unable to identify datasets to quantify the role of these services. Additionally, population displacement data were on an annual temporal scale and did not cover the entire country, thus limiting our ability to have precise quantitative estimates of the contribution of movement as shown with cell phone data or theoretical models in other settings.^36^, ^37^ Despite these limitations, trends of mass migration and spread of cholera were largely consistent.

Cholera has not been reported in South Sudan from November, 2017, up to the time of last editing this manuscript (Nov 16, 2020), which might be explained by a combination of natural and vaccine-derived immunity, reduced displacement, and perhaps improved sanitary conditions. Estimated water and sanitation indicators remain poor in South Sudan; only 36·7% of the population had access to improved water and 9·9% had access to improved sanitation in 2017.^2^ South Sudan will probably continue to remain at risk of introductions of cholera from other countries in the region, which could then lead to large-scale spread. Broad use of oral cholera vaccine combined with local WASH interventions might provide a stopgap over the coming years to block transmission while much-needed investments in water and sanitation are made. However, in South Sudan, peace is probably the first prerequisite to long-lasting cholera prevention.

Through better understanding of how cholera spreads and the complex roles of human movement, climate, and cholera-specific interventions, it might be possible to plan for quicker and appropriately targeted cholera control measures in future cholera epidemics in South Sudan and beyond. Such advances in humanitarian emergencies might be crucial to achieving progress towards the 2030 cholera goals of reducing cholera as a public health threat.
